# Cognitive deficits in psychiatric disorders: Current status

**DOI:** 10.4103/0019-5545.31613

**Published:** 2006

**Authors:** J.K. Trivedi

**Affiliations:** Professor, Department of Psychiatry, King George Medical University, Lucknow 226006, Uttar Pradesh, e-mail: drjktrivedi@sify.com, drjktrivedi@sancharnet.in, jktrivedi@hotmail.com, jitendra.trivedi@gmail.com

**Keywords:** Cognitive deficits, neurocognition, treatment of psychiatric disorders

## Abstract

Cognition denotes a relatively high level of processing of specific information including thinking, memory, perception, motivation, skilled movements and language. Cognitive psychology has become an important discipline in the research of a number of psychiatric disorders, ranging from severe psychotic illness such as schizophrenia to relatively benign, yet significantly disabling, non-psychotic illnesses such as somatoform disorder. Research in the area of neurocognition has started unlocking various secrets of psychiatric disorders, such as revealing the biological underpinnings, explaining the underlying psychopathology and issues related to course, outcome and treatment strategies. Such research has also attempted to uproot a number of previously held concepts, such as Kraepelin's dichotomy. Although the range of cognitive problems can be diverse, there are several cognitive domains, including executive function, attention and information processing, and working memory, which appear more frequently at risk. A broad range of impairment across and within the psychiatric disorders are highlighted in this oration. The oration summarizes the studies investigating cognitive processing in different psychiatric disorders. I will also discuss the findings of my own research on neurocognitive deficits in mood disorders, schizophrenia, obsessive–compulsive disorder, somatoform disorder, including studies on ‘high-risk’ individuals. Tracing the evaluation of neurocognitive science may provide new insights into the pathophysiology and treatment of psychiatric disorders.

## INTRODUCTION

To begin with, I am immensely thankful to the Indian Psychiatric Society for bestowing the honour on me—to deliver this prestigious oration. This oration has enabled me to pay my tributes to Late Dr DLN Murthy Rao—the renowned psychiatrist who has helped many in the Indian psychiatry to reach the present height. I wish to pay my gratitude and sincere thanks to my teachers Late Professor B.B. Sethi, Professor A.K. Agarwal, Professor Narottam Lal, Professor S.C. Gupta and Shri P.K, Sinha for their blessings and support. I feel extremely privileged as I stand before you and focus on one of the most interesting topic in psychiatry—‘Cognition’.

Cognitive psychology has become an important area of research in a number of psychiatric disorders, ranging from severe psychotic illness such as schizophrenia to relatively benign, yet significantly disabling, non-psychotic illnesses such as somatoform disorder. Research in the area of neurocognition has started unlocking various secrets of psychotic disorders, such as revealing the biological underpinnings, explaining the underlying psychopathology and issues related to course, outcome and treatment strategies.

This oration summarizes the studies investigating cognitive processing in different psychiatric disorders, with an emphasis on recent concepts. I will also discuss the findings of my team's research on neurocognitive deficits in mood disorder (including a high-risk group study), schizophrenia, obsessive–compulsive disorder and somatoform disorder carried out in the Department of Psychiatry, K.G. Medical University, Lucknow. Tracing the evolution of neurocognitive science may provide new insights into the pathophysiology and treatment of psychiatric disorders.

## COGNITION

Cognition in a broad sense means information processing. It denotes a relatively high level of processing of specific information including thinking, memory, perception, motivation, skilled movements and language. The hippocampus contains the neural circuitry crucial for cognitive functions such as learning and memory. It refers to the perceptual and intellectual aspects of mental functioning. Among the specific functions that may be assessed in determining the intactness or adequacy of cognition are orientation, the ability to learn necessary skills, solve problems, think abstractly, reason and make judgements, the ability to retain and recall events, mathematical ability and other forms of symbol manipulation, control over primitive reactions and behaviour, language use and comprehension, attention, perception and praxis.^1^ Cognitive deficits may result in the inability to:

1. pay attention
2. process information quickly
3. remember and recall information
4. respond to information quickly
5. think critically, plan, organize and solve problems
6. initiate speech

The following questions need to be addressed in relation to psychiatric disorders and cognitive impairment:

1. Is there anything specific about the profile of cognitive impairment in these disorders?
2. How do these impairments relate to the underlying psychopathology/neuroanatomy of these disorders?
3. Do these cognitive impairments vary over a course of time?
4. What impact do these impairments have in terms of treatment implications?

**Fig 1:**
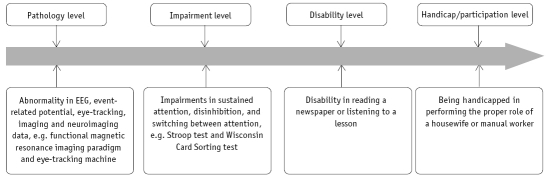
Levels of disease process and types of assessments-relationship

Cognitive deficits have a relationship with different levels of the disease process, as depicted in Fig 1^2^ Before discussing specific cognitive deficits in different psychiatric disorders, the following cognitive domains need to be elucidated.

## WORKING MEMORY

Working memory (WM) function is thought to be sustained by a network of temporary memory systems. It plays a crucial role in many cognitive tasks, such as reasoning, learning and understanding. It refers to the ability to hold the stimuli ‘online’ for a short time, then either use it directly after a short delay or process or manipulate it mentally to solve cognitive and behavioural tasks. WM involves active rehearsing, processing and manipulation of information. WM seems to depend on the function of the prefrontal cortex.^3^

## EXECUTIVE FUNCTION

Executive function (EF) refers to the ability to use abstract concepts, to form an appropriate problem-solving test for the attainment of future goals, to plan one's actions, to work out strategies for problem-solving, and to execute these with the self-monitoring of one's mental and physical processes. Executive skills are most important in dealing with novel or complex situations. Physiologically, EF is linked to the cortical–subcortical circuits and frontal lobes.^4^

## ATTENTION AND INFORMATION PROCESSING

Attention refers to the ability to identify relevant stimuli, focus on these stimuli rather than others (selective attention), ability to perform a task in the presence of distracting stimuli (focused attention), sustain focus on the stimulus until it is processed (sustained attention or vigilance), and allow for the transfer of the stimulus to higher-level processes. The Continuous Performance Test (CPT) is commonly used for measuring attention.

Cognitive deficits have been an area of research in many psychiatric disorders, the ‘prototype’ work being in schizophrenia. I will begin with the review of the research on cognitive deficits in schizophrenia.

## COGNITIVE DEFICITS IN SCHIZOPHRENIA

The understanding of the fundamental deficits in schizophrenia comes full circle as it begins to be accepted that cognitive dysfunctions play a central role in the illness, as Kraepelin and Bleuler had suggested. Cognitive deficits are a core feature of schizophrenia which

—may precipitate psychotic and negative symptoms;^5^

—are relatively stable over time, with progressive deterioration after the age of 65 years in some patients;^6^

—persist on the remission of psychotic symptoms;^7^

—are related to but separate from negative symptoms;^8^,^9^ and

—determine the functional impairment characteristics of patients with this disorder.^10^

An important domain of schizophrenia that appears closely related to functional outcome involves cognitive deficits.^11^,^12^ The range of cognitive deficits is broad and includes problems in perception, attention, memory and problem-solving.^13^

WM deficits in schizophrenia reflect hypofunction of the prefrontal cortex (PFC). Neuropsychological and imaging studies suggest that the WM system is of a limited capacity in patients with schizophrenia.^14^–^17^ WM deficits have been found to correlate significantly with formal thought disorder,^18^ and may result in loose association and derailment.^19^ Deficits in strategic long-term memory (e.g. free recall, memory for temporal order) could be accounted for by deficits in WM.^20^

Patients with schizophrenia also show deficits in measures of EF.^21^,^22^ Severity of negative symptoms such as affective flattening, alogia, social withdrawal or avolition has been found to be associated with poor performance on measures of EF.^23^ Performance on the measures of executive functioning has been found to be linked to insight of illness^23^,^24^ and hence to poor medication compliance, self—injurious behaviour and assaultiveness.^25^–^27^

Deficits in attention and information processing might be central to schizophrenia because these can contribute to deficits in EF and WM.^28^ Attention deficits are also trait and vulnerability markers seen during remission^29^ in children of schizophrenic parents^30^ and individuals with schizotypal personality.^31^ Attention deficits have been found to be robustly associated with deficit syndrome.^32^ Distractability has been associated with higher levels of formal thought disorder.^33^

Although there appears to be a group of patients who are impaired only minimally,^34^–^36^ most patients are characterized as having at least some impairment across a number of domains. More specific deficits in schizophrenia occur within the context of diffuse cognitive impairment—especially in areas of verbal episodic memory and vigilance.^37^–^39^

Some deficits including verbal memory and learning, visual memory, abstraction, attention and language abilities have been found even in untreated, first-episode patients.^40^–^43^

Cognitive impairments differ according to the clinical symptomatology—the deficits may be related more with disorganized and negative symptoms and less with psychotic symptoms.^44^–^48^ There appears no pathognomonic neuropsychological profile in schizophrenia, likely due in part to aetiological heterogeneity within the disorder.

Cognitive deficits and functional impairment manifest a specific pattern of relationship in schizophrenia.

**Table:** 

Functional domain	Cognitive correlates
Social function	Declarative memory, vigilance, EF
Occupational functioning	EF, declarative memory, WM, vigilance
Independent functioning	EF, declarative memory, WM

EF: executive function; WM: working memory

## IMPROVING COGNITION IN SCHIZOPHRENIA

Newer antipsychotic medications have been shown to have neurocognitive advantages over conventional antipsychotic medications.^49^–^52^ Atypical antipsychotic medications differ in their profiles of cognitive efficacy; for example, risperidone shows relatively greater improvement in memory; olanzapine causes greater improvement in processing speed.^53^,^54^ Determining and characterizing the cognitive profile of each atypical antipsychotic medication is an important task, as this information could be used to target the cognitive problems of individual patients.

Although atypical antipsychotic medications appear to have some benefit on cognitive function, further efforts are required to improve cognitive function. Such methods may be pharmacological and psychological such as cognitive remediation. Cognitive remediation targets three domains—EF, attention and memory, and there is evidence for gains in performance across all three domains,^55^ particularly for WM.^56^ Adjunctive pharmacological interventions, with some reported effectiveness include nicotinic treatment for attentional difficulties,^57^ tandospirone for memory problems,^58^ donepezil^59^–^61^ and NMDA receptor stimulating agents.^62^

Such newer psychopharmacological and neuropsychological remediation programmes may provide clinicians with a variety of means to improve cognitive and social functioning in schizophrenia.

## COGNITIVE IMPAIRMENTS IN MOOD DISORDERS

Neurocognitive deficits are present in mood disorders. In major depression, cognitive impairment can be severe and global, mimicking dementia.^63^ In the acute phase of bipolar disorder, impairment of cognition may progress to a stuporous state. Cognitive deficits in mood disorders include impaired performance in tests of attention, EF and memory. Increased cognitive dysfunction is associated with greater severity of symptoms. Owing to the presence of cognitive deficits even during the euthymic/remitted states, it is suggested that certain cognitive deficits are fundamental trait characteristics. Impairment of WM,^64^ sustained attention,^65^,^66^ focusing-execution,^67^ abstract reasoning and visuomotor skills,^68^ verbal memory,^69^,^70^ verbal fluency,^71^ visuospatial ability,^72^ have all been reported, even in the euthymic phase of the illness.

The deficits have been shown to correlate with both the number of affective episodes and the overall duration of illness.^65^,^71^,^73^,^74^,^76^ Performance on memory and executive tasks were most likely to correlate with illness episodes.

The relationships between mood and cognition are dynamic ones, with components that are trait-dependent and others that are state-dependent. Because of their relatively static nature, trait characteristics of cognitive and neurological manifestation may provide insights into core brain anomalies that give rise to severe mood disorders.

## COGNITIVE DEFICITS: A CHALLENGE TO KRAEPELIN'S DICHOTOMY

In 1896, Kraepelin proposed the classification of the psychoses into dementia praecox and manic–depressive insanity. The latter was characterized by an episodic course and benign prognosis, and formed the basis of the concept of bipolar disorder. Recent investigations, which demonstrate neuropsychological impairment in euthymic bipolar patients, have posed a challenge to Kraepelin's dichotomy.

Schizophrenia and mood disorders have significant overlap in genetic and neuroimaging studies as well.^77^,^78^ In contrast to the concepts of Kraepelin, bipolar disorders may be chronic; hence, long-term therapy may be needed for bipolar disorder, as is true for schizophrenia.

## COGNITIVE DEFICITS IN BIPOLAR DISORDER AND SCHIZOPHRENIA: A COMPARISON

Similar cognitive profiles have been reported in patients with bipolar disorder and schizophrenia, but the severity of impairment appears greater in schizophrenia.^79^–^81^ There is now substantial evidence that cognition is a good predictor of functional outcome in schizophrenia as well as bipolar-I disorder.^12^ Controversies, however, exist regarding the specificity of domains or the generalizability.

Comparisons of cognition between these patient groups are problematic due to the fact that differences in illness characteristics and current symptoms are not always assessed and may confound neuropsychological test performance.^80^–^83^

A major limitation is differing medication regimens. Patients with bipolar disorder usually receive mood stabilizers (e.g. lithium, anticonvulsants) whereas patients with schizophrenia often take antipsychotic medications. Also, it is impossible to assess the degree of the patient's cognitive impairment if studies fail to include a normal control group.^83^,^84^

In a study conducted at our centre (tertiary-care psychiatric hospital), 15 stable maintained schizophrenic patients and 15 euthymic bipolar-I patients attending the outpatient clinic were compared with each other as well as with 15 age- and education-matched controls. Stable schizophrenic patients were clinically assessed using the Positive and Negative Syndrome Scale for Schizophrenia (PANSS) and Hamilton Depression Rating Scale (HDRS) while euthymic patients were clinically assessed using the Young Mania Rating Scale (YMRS) and HDRS. Neurocognitive assessments were done using the WCST, Spatial Working Memory Test (SWMT) and CPT—all are computer-based. Stable schizophrenic patients performed poorly on all the neurocognitive parameters as compared with both controls and bipolar euthymic patients. Euthymic bipolar patients showed a significant difference in the domain of EF compared with normal controls, while the schizophrenic and bipolar euthymic patients were comparable. Thus, our study suggested that bipolar patients in the euthymic/remitted phase also have some types of cognitive processing deficits. Patterns of cognitive disturbances in tasks of EF are similar in both the groups but are quantitatively more marked in schizophrenia. Euthymic bipolar subjects showed significant impairment only on EF when compared with the control group.^85^

## COGNITION DURING REMISSION IN BIPOLAR DISORDER

Cognitive deficits in attention, EF and memory have been recorded in bipolar patients. Cognitive deficits persist during remission and indicate some type of cognitive processing deficits that may be fundamental trait characteristics. Examining the deficits during remission may help in a better understanding of the course of non-affective symptoms associated with mood disorders.

Although cortical dysfunction undoubtedly plays a role in the development of abnormal mood states, the use of neuropsychological assessments as a tool to probe such dysfunction is limited by a number of non-specific factors. Poor performance on neuropsychological tests may be due to reduced motivation, non-cooperation or failure to remain engaged in a formal test setting.^75^ Recently, cognitive assessments have been performed in bipolar patients when they are euthymic. Studies of this nature aim to identify trait-dependent rather than state-dependent neuropsychological deficits, which may be indicative of underlying neurobiological disturbances associated with the pathophysiology of bipolar disorder. Results from various studies are not consistent and therefore raise questions about the generalization of the findings. Some studies fail to control for differences in age, gender and premorbid intelligence between patients and control subjects.^86^ Euthymia is often poorly defined.^73^,^83^ Different neuropsychological measures used by investigators to study neurocognitive functions of bipolar disorder further limit the interpretability and generalizability of the findings. Yet, patterns that emerge from the literature demonstrate the fact that neurocognitive impairments form an important clinical component of bipolar illness and are not merely epiphenomena.

A high incidence of psychosocial difficulties has been reported during remission, which raises questions about so-called complete recovery. Patients in either acute manic or euthymic states have demonstrated significant impairment on the Stroop test, a test for EF.^87^

Zubeita *et al.*^88^ found that euthymic patients did not show significant impairment on CPT, however, on WCST patients committed more errors than healthy controls. The same study also showed impairment in verbal memory, but not in non-verbal memory. Subjective memory complaints are often reported during the euthymic state in bipolar patients. Impairment of memory has been reported during the depressed, manic and euthymic phases of the illness. Impairments in story recitation^88^ and word list recall^69^ have been recorded in the euthymic phase.

In a study conducted at our centre on 30 patients meeting the DSM-IV criteria for bipolar affective disorder, currently euthymic, were assessed using WCST for EF, CPT for performance on continuous tasks administering and SWMT for spatial working memory. Fifteen controls matched for age and sex were also assessed. All the patients and controls were educated at least up to standard VIII. The patients’ performance was impaired on WCST, implying poor EF as compared with controls, and indicating residual cognitive deficits. The performance on tests of attention, concentration and memory was not significantly different from that of the matched controls.^89^

## HIGH—RISK STUDIES: EVIDENCE FOR TRAIT MARKERS

Neurocognitive deficits in individuals with bipolar disorder are not merely the product of affective symptomatology or medication use, but are reflective of premorbid developmental abnormalities. Support for this theoretical position is derived from a variety of methodological approaches: twin studies, comparison of cases with positive and negative family histories, retrospective analyses of premorbid function and assessment of unaffected biological relatives. ‘High-risk’ individuals are those who have an increased genetic risk for bipolar disorder (e.g. monozygotic twins, first-degree relatives) but without outright expression of the illness. Studies on these individuals are unlikely to be confounded by medication or effects of chronicity of illness. Few studies of bipolar disorder have employed a high-risk paradigm model (unlike large number of studies on schizophrenia).

Keri *et al.*^90^ showed that the unaffected relatives of bipolar patients showed a greater degree of verbal recall difficulties than a group of unrelated controls. Chowdhury *et al.*^91^ suggested deficits in the executive control of WM to be a trait deficit. Zalla *et al.*^92^ found that the unaffected relatives of bipolar patients performed poorly on the Stroop test, i.e. a dysfunction of anterior cingulate cortex. McDonough–Ryan *et al.*^93^ found significant impairment on executive and non-verbal intelligence test—associated tasks in children with at least one parent with bipolar disorder.

However, not all studies of ‘at risk’ relatives of bipolar patients have reported the presence of cognitive deficits.^94^–^96^ The plastic changes associated with a mood-driven disturbance of attention may adversely affect cognition particularly in the acute stage of the bipolar illness. Analysis of executive-type cognitive traits may constitute a key endophenotype (vulnerability marker) for genetic studies of bipolar disorder.^97^

We conducted a study to assess neurocognitive functions in 10 first-degree healthy relatives of patients with bipolar disorder and compared them with 10 age-, gender- and education-matched healthy controls with no family history of any neuropsychiatric disorder. In this study, EF assessed using WCST showed that healthy first-degree relatives of patients with bipolar disorder completed less categories and committed more perseverative errors than did normal controls, i.e. they had impaired EF.

On CPT, attention and concentration abilities were found to be significantly impaired; and first—degree healthy relatives of bipolar patients committed more mistakes (errors of commission), had more missed responses (errors of omission) and took more time to respond then normal controls.

For spatial working memory on SWMT, healthy first-degree relatives of patients with bipolar disorder were found to be significantly impaired (scored less correct responses) at 0 sec delay (i.e. error in recognition) but no significant differences were reported between subjects (relatives) and controls at 20 sec delay (i.e. no error in recall). No significant differences were seen regarding non-adjacent errors at 0 sec and 20 sec delays.^98^

The evidence suggests that the presence of cognitive dysfunction in bipolar affective disorder is a core and enduring deficit of the illness. The association between cognitive impairment and the number of affective episodes suggests that each affective episode is not biologically benign and early diagnosis followed by active treatment could potentially reduce cognitive morbidity. Future studies are required employing a variety of study designs such as the use of high-risk groups and first-episode patients, in conjunction with longitudinal assessments. Understanding the mechanism that underlies mood and cognition may help to devise better treatment options to address dysfunctions during euthymia.

## COGNITION IN DEPRESSION

Numerous studies have demonstrated the presence of neuropsychological deficits in actually depressed patients with verbal and visual memory as well as EF.^99^–^101^ The decrement in cognition has been attributed to reduced motivation, attenuated attentional capacity, impaired concentration, intrusive thought and slowness. Cognitive deficits are more pronounced in melancholic than non-melancholic depression.^102^ Prominent cognitive disturbances may be one of the presenting symptoms of depression. Drevets *et al.*^103^ showed reduced blood flow to the subgenual area of the prefrontal cortex in bipolar and unipolar depression. Core cognitive deficits may be present in unipolar depression which is independent of the depressed state.^87^

In the acute phase of depression, patients produce more errors of attention compared with matched controls.^104^ Austin *et al.*^105^ showed impairment in EF in the Trail Making Test, part B, which worsened with the level of depression. Verbal memory impairment, such as story recitation and word list recall, has been reported.^88^,^105^

Non-verbal memory has also been reportedly impaired in depressed patients.^106^ Implicit non-declarative memory has not been found to be impaired in depressed patients.^107^

We carried out a study at our centre on 30 patients with depression and 15 matched controls, and compared their performance on the tests of EF, attention–concentration and WM. The findings of the study suggest that depression induces significant impairment in the abilities of sustained attention as shown by the fewer correct responses and more missed responses (errors of omission), and this impairment increases with severity. The number of wrong responses, however, was similar in controls and patients (errors of commission). Reaction time also increases due to depression, which further increases with severity.

Significant impairment in spatial working memory after a delay (error in recall) is seen as compared with controls, its extent, however, is similar across the variable severity.

Patients performed similar to controls at 0 sec (recognition). On WCST, executive dysfunction was significant in the patient group; more severe illness was associated with greater impairment in EF.^108^

## COGNITIVE DEFICITS IN OBSESSIVE—COMPULSIVE DISORDER (OCD)

A recent area of research has been the characterization of neuropsychological deficits in patients with OCD. This has contributed to the understanding of biological underpinnings of the illness. Cognitive deficits could be functioning as an intermediate variable between neurobiological abnormalities and OCD symptoms. Reductions in social competence and the capacity for independent living and vocational success may be the result of neurocognitive compromise.

EF deficits have been seen in several studies among OCD patients.^109^–^112^ These EF deficits may explain partly the performance difficulties seen in patients with OCD in other cognitive domains.^113^ Okasha *et al.*^114^ suggested that patients with OCD are unable to disregard irrelevant stimuli and may become overwhelmed by this information.

Visuospatial and visuoconstructional deficits are among the most consistent findings in neuropsychological assessment studies of patients with OCD.^114^,^115^

OCD patient groups have shown impairment on numerous tests of non-verbal memory including visual reproduction and delayed recognition of figures, maze learning and intermediate and delayed figure copying. Most studies suggest that encoding and retrieval are impaired in OCD, while storage of information remains intact. Some studies suggest that deficits in encoding of new information are primarily responsible for these performance problems.^111^,^116^ Savage *et al.*^117^ indicate that retrieval is also faulty, while storage is intact. Deckersbach *et al.*^118^ reported that OCD patient groups have deficits in the implicit memory domain of procedural learning in dual task condition. This finding was interpreted as consistent with frontostriatal dysfunction.

Patients with OCD often function remarkably well in their daily lives, despite severe symptomatology and cognitive difficulties, which are apparent only on specific testing. In contrast to non-verbal memory deficits, verbal memory is generally preserved in studies of patients with OCD.^115^,^119^,^120^ Patients with OCD demonstrate normal general intelligence and language abilities. Different subtypes of OCD may have varying neuropsychological deficit profiles.^121^

In a study we aimed to evaluate the EF, spatial working memory and performance on continuous tasks of 30 patients with OCD compared with 30 normal matched healthy controls, and to observe the effect of severity and duration of psychopathology on them.

Findings of the study suggested that OCD induces significant impairment in the EF as lesser categories were completed and more perseverative errors were committed by patients, and that the degree of impairment increases with the severity of the illness.

OCD induces significant impairment in sustained attention abilities, as shown by fewer correct responses and more missed and wrong responses; the degree of impairment does not increase with severity.

Reaction time was, however, not significantly different between the patients and controls.

Significant impairment in spatial working memory was seen as compared controls after a delay (error in recall); its extent also increased with increasing severity. The patients performed similar to controls at 0 sec (recognition).

No effect was found of the duration of illness on performance in any of the domains.

Ascertaining whether cognitive impairment is a function of the present disease state or a long-term stable trait has both heuristic and clinical implications.^122^

## COGNITION IN SOMATOFORM DISORDER

Somatoform disorder includes somatic, psychopathological and neuropsychological symptoms. Cognitive complaints reported frequently by patients include poor concentration, decreased memory for recent events and poor word-finding abilities.^123^ About 50%–85% of patients with somatoform/chronic fatigue syndrome (CFS) report cognitive problems, which contribute considerably to their social and occupational dysfunction.^124^,^125^

Studies have revealed that impaired attention–concentration abilities and spatial working memory in patients of somatoform disorder^126^–^129^ also reported significantly slow responses. Cognitive problems in severe somatization provide a challenge to future research as well as diagnostic and treatment options. Early identification of the cognitive dysfunction in somatoform disorder, namely slowed processing speed, impaired working memory, poor learning information, poor set shifting and planning ability, would enable us to offer appropriate advice on coping with these and provide considerable benefit to patients.

Niemi *et al.*^130^ showed that patients of somatoform disorder performed at a lower level than controls in tests involving semantic memory, verbal episodic memory and visuospatial tasks, and the fact that they were slower in attentional tasks may further suggest that somatization is associated with brain dysfunction, especially impaired anterior control of attention and memory.

In a study between 1 May and 31 July 2004, we assessed 20 patients who met the ICD-10 criteria for somatoform disorder by using the test for EF on WCST, performance on continuous task on CPT and spatial working memory on SWMT. Fifteen controls (age-, education- and gender-matched) were also assessed. All the patients and controls were educated at least up to standard VIII. The patients’ performance was impaired on WCST implying poor EF as compared with controls. The performance on tests of attention-concentration was also significantly poorer than that of the controls. On the SWMT, however, significant difference was found only in delayed retrieval and not in immediate recall. The study suggested that enduring cognitive deficits in EF, attention-concentration and memory may be contributing to the poor psychosocial functioning in patients with somatoform disorders.^131^

## COGNITIVE DEFICITS IN OTHER PSYCHIATRIC DISORDERS

Studies related to neuropsychological dysfunction are invading many other psychiatric disorders as well. Some important psychiatric disorders are discussed below.

### Cognitive deficits in borderline personality disorder (BPD)

Neuropsychological and psychopathological factors in personality disorders seem to be related thus, they challenge the assumption that personality traits are responsible for the behavioural and emotional experiences associated with BPD. Studies have revealed poor decision-making skills.^132^,^133^ Burgees^134^ revealed a link between attention and memory impairment and self-injury. O'Leary *et al.*^132^ showed that although verbal memory skills of BPD patients were impaired, their memory improved with the use of cues. Thus, clinicians might make use of cueing strategies when working with these patients. Paris *et al.*^135^ found significant impairment on tests of frontal lobe and executive functions in children with BPD, and also showed inconsistent levels of attention, poor orientation to task and slower reaction on CPT. In fact, suicide risk in BPD patients has been linked to cognitive functioning and not to level of depression.^134^

### Cognitive deficits in attention deficit/hyperactivity disorder (ADHD)

In general, poor performance on tests of EF, sustained attention and memory tend to be the most common neuropsychological deficits reported in children and adults with ADHD. There is little evidence for deficits in basic motor, visuospatial or sensory functioning in ADHD, with the possible exception of olfactory processing.^136^,^137^ In particular, converging evidence points to a prominent disturbance of EF.^136^,^138^,^139^ Various execution functions that have been studied in relation to ADHD are: cognitive flexibility, initiation, interference control, planning and organization, response inhibition, self-monitoring and WM. Further research using cognitive tasks assessing EF in combination with functional imaging techniques will provide insights into the aetiology of the disorder.

### Cognitive impairments in substance abuse

Cognitive dysfunctions have been demonstrated following substance abuse. These functions include mental activities that involve acquiring, storing, retrieving and using information. These cognitive functions could play an important role in the development of the addictive process and rehabilitation of substance abusers. Prochaska *et al.*^140^ postulated that cognitive skills are critical for drinking behaviour change. Memory and EF are likely to influence the execution of skills that are implicated for both motivating and sustaining drinking behaviour change. Blume *et al.*^141^ showed that an explicit memory process may have utility for predicting readiness to change drinking behaviour. Lyvers *et al.*^142^ showed that opioid dependence such as alcohol addiction is associated with cognitive dysfunctions. Newly detoxified alcoholics exhibit relatively intact verbal and general intellectual abilities, but impaired non-verbal abilities.^143^,^144^ Deficits exist in novel problem-solving, abstract reasoning, and learning and recalling information.^144^,^145^ Deficits in perceptual–motor abilities, abstract reasoning, and non-verbal learning and memory can persist for months or years.^145^–^147^ Neuropsychological test findings with substance-dependent populations also might influence the design of treatment programmes.^143^,^148^

## CONCLUSION

Although the range of cognitive problems can be diverse, there are several cognitive domains, including executive, attentional and memory, that appear most frequently at risk. Without a doubt, there is more to be learned about the specificity of cognitive impairment across disorders, the relationship of these deficits with the underlying psychopathology and neuroanatomy of these disorders, and the impact of the impairments on treatment implications. Early identification of these dysfunctions would provide considerable benefit to patients and suggest ways of coping with these dysfunctions.
